# Effect of cholesterol re-supplementation and atorvastatin on plaque composition in the thoracic aorta of New Zealand white rabbits

**DOI:** 10.1186/s12872-020-01703-x

**Published:** 2020-09-17

**Authors:** G. A. Bonaterra, K. Bender, B. Wilhelm, H. Schwarzbach, S. Metz, O. Kelber, D. Weiser, J. Metz, R. Kinscherf

**Affiliations:** 1 Department of Medical Cell Biology, University of Marburg, 35032 Marburg, Germany; 2 Department of Anatomy and Cell Biology III, University of Heidelberg, 69120 Heidelberg, Germany; 3 Department of Radiology, Technical University, 81675 Munich, Germany; 4 Steigerwald Arzneimittelwerk, 64295 Darmstadt, Germany

**Keywords:** Apoptosis, Atherosclerosis, Atorvastatin, Macrophage, Hypercholesterolemic rabbits, Re-supplementation

## Abstract

**Background:**

Effects of re-supplementation of a cholesterol-enriched diet (CEDrs) on size, cholesterol content and morphology of already existing plaques are not known to date.

**Methods:**

A group of rabbits received standard chow (SC) for 6 weeks (“negative control”; for plasma lipid measurements only). Group I-IV received 2% CED (induction) for 6 weeks; thereafter, groups II-IV have been fed a SC (= cholesterol withdrawal) for 68 weeks. Afterwards, feeding of groups II-IV was continued as follows: Group II - 10 weeks SC, group III - 4 weeks 0.5% CED (~re-supplementation), afterwards 6 weeks SC (~withdrawal again); group IV - 4 weeks 0.5% CED (re-supplementation) + atorvastatin (2.5 mg/kg body weight/day), afterwards 6 weeks SC (~withdrawal again) + atorvastatin. Plasma lipids, but also plaque size, morphology and cholesterol contents of thoracic aortas were quantified.

**Results:**

After CEDrs, plasma cholesterol levels were increased. However, after withdrawal of CEDrs, plasma cholesterol levels decreased, whereas the cholesterol content of the thoracic aorta was increased in comparison with the group without CEDrs. Plaque size remained unaffected. Atorvastatin application did not change plasma cholesterol level, cholesterol content of the thoracic aorta and plaque size in comparison with the group without drug treatment. However, atorvastatin treatment increased the density of macrophages (MΦ) compared with the group without treatment, with a significant correlation between densities of MΦ (Mac-1^+^) and apoptotic (TUNEL^+^; TP53^+^), antigen-presenting (HLA-DR^+^) or oxidatively stressed (SOD2^+^) cells.

**Conclusions:**

In rabbits with already existing plaques, CEDrs affects plaque morphology and cellular composition, but not plaque size. Despite missing effects on plasma cholesterol levels, cholesterol content of the thoracic aorta and size of already existing atherosclerotic plaques, atorvastatin treatment transforms the already existing lesions to a more active form, which may accelerate the remodelling to a more stable plaque.

## Background

The typical nutrition in Western societies consists of highly processed and palatable foods, with high contents in refined sugar, fat, oil, and meat. These foodstuffs are daily consumed in industrial countries and have been called “Western diet”. There is no doubt that the “Western diet” is closely associated with atherosclerosis, characterized by hardening of the arteries, elevated blood lipids, i.e. values that reflect the quality and quantity of fat in an individual’s eating habit. For this reason, the role of nutrition in the prevention of cardiovascular diseases (CVD) has been extensively reviewed. Strong evidence exists, showing that dietary factors may directly influence atherosclerosis or through effects of traditional risk factors, e.g. plasma lipids, blood pressure, or glucose [1]. In this regard, feeding cholesterol to rabbits is a widely used model for experimental atherosclerosis research. Already in 1913, Anitschkow and Chalatow demonstrated that it was just cholesterol that causes these atherosclerotic changes in the arterial intima of rabbits, which was very similar to human atherosclerosis [2]; however, these about 100 years-old findings have been also confirmed by most recent publications [3, 4]. The first prospective interventional study in which a significant plaque diminution was observed in cholesterol-fed rabbits was performed in the 1950s [5]. Moreover, studies in non-rodent and murine models have most recently shown that atherosclerosis is a reversible process [6]. Using an animal model for atherosclerosis, it has been most recently found, that after atherosclerosis induction by a cholesterol-enriched diet (CED), lipid-lowering diet can lead to plaque stabilization associated with increased storage of collagen in the fibrous cap, a signature of a beneficial remodeling of atheromatous lesions in rabbits [6]. Additionally, in experimental atherosclerosis, using male New Zealand White (NZW) rabbits, withdrawal of CED and statin therapy revealed evidence of plaque stabilization as shown by decreased macrophage (MΦ) infiltration and apoptosis, as well as an increase of smooth muscle cell (SMC) content [7]. Hence, key players in the pathogenesis of atherosclerotic plaque progression and regression is MΦ, which also represent a risk factor for plaque rupture [3, 8] or remodeling [9–11].

Furthermore, apoptosis is related to plaque instability and dietary modification. Also, histomorphological data suggest that apoptosis of MΦ contributes to the enlargement of the lipid core, which occurs on sites of plaque rupture and probably initiates events leading to rupture [7]. In general, vulnerable plaques are characterized by a thin fibrous cap, a large lipid core, an abundance of MΦ and T lymphocytes, as well as a reduced density of SMC [12, 13], leading to initiation of plaque rupture [14–16]. Vice versa, the reduction in total plaque lipid burden, resolution of inflammation and neutralization of matrix metalloproteinase expression contribute to plaque stabilization [7, 17]. Moreover, plaque shrinkage is a coordinated process that involves the depletion of foam cells and extracellular cholesterol stores, i.e. a gradual decline of MΦ numbers through enhanced emigration from the plaque, and replacement of inflammatory MΦ with anti-inflammatory phagocytes, being associated with the removal of necrotic material, tissue healing and lesion stabilization [6, 11, 16, 18]. Additionally, the reduction of the consumption of calories and/or the decrease of serum cholesterol concentration by using the lipid-lowering medication, most often statins, is frequently used because cardiovascular events/morbidity and mortality in patients are decreased [19–21]. Besides that, statin therapy is associated with the prevention of acute coronary syndrome [7]. It has been demonstrated, that a low-dose combination of atorvastatin and valsartan, used for the treatment of high blood pressure, protected the thoracic aorta wall in guinea pigs from atherogenic diet-induced lesions as well as nanoparticle of hyaluronan-atorvastatin conjugate to increase the bioavailability [22, 23].

However, during plaque development, the composition of atherosclerotic plaques seem to be more relevant for (in) stability than the size of a plaque. Thus, in patients with already existing atherosclerotic lesions, the crucial question remains: How is the plaque phenotype affected by changes of a period of interruption of a lipid-rich diet to a low-fat diet accompanied with or without the treatment with lipid-lowering medication?

Therefore, the purpose of our study using an experimental model of atherosclerosis, i.e. rabbits with previously developed atherosclerotic lesions was to investigate the effects of CED re-supplementation (CEDrs) with or without statin treatment, with special attention to plaque composition.

## Methods

### Animal procedures

**Fig. 1:**
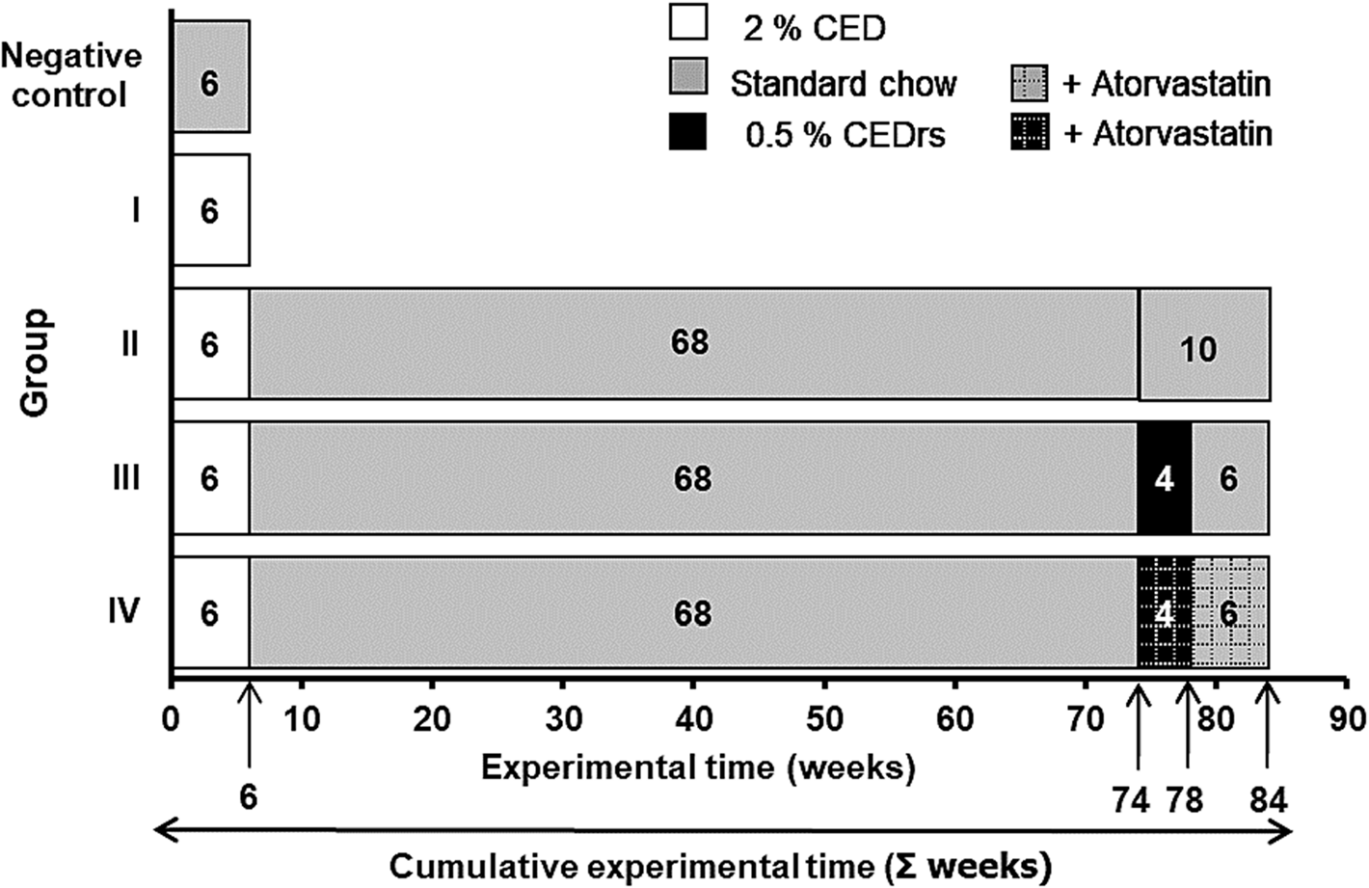
Time schedule of the dietary interventions used in this study. Cholesterol-enriched diet (CED); CED re-supplementation (CEDrs) and control group after feeding for six week with standard chow. Black arrow, “6 weeks”, indicates the end point of the CED induction period. Black arrow, “74 weeks” indicates the end point of the 68 weeks withdrawal and the initiation of the 4 weeks CEDrs. Black arrow, “78 weeks”, indicates the end of 4 weeks CEDrs with or without atorvastatin treatment. Black arrow, “84 weeks”, indicates the endpoint of the study

Male NZW rabbits, weighing 3–5 kg were purchased from Charles River Farm (Sulzfeld, Germany). An approval/permission from the farm owner to use the animals was not necessary. Animals were housed one in each cage under the same conditions, with dark-light cycles of 12 h and constant temperature of 24 ± 2 °C with ad libitum access to food and water in boxes of 55 × 30 cm and 25 cm high. The animals had olfactory, visual, and auditory contact with the other rabbits. Animals were distributed randomly into different groups. A group of rabbits (*n* = 8) received standard chow (SC) for 6 weeks (“negative control”; for plasma lipid measurements only). Group I (*n* = 8) to group IV received 2% CED (induction) for 6 weeks; thereafter, groups II-IV have been fed a SC (= cholesterol withdrawal) for 68 weeks (Fig. 1). Afterwards, feeding of groups II-IV was continued as follows: Group II (*n* = 5) - 10 weeks SC; group III (*n* = 5)- 4 weeks 0.5% CED (~re-supplementation), afterwards 6 weeks SC (~withdrawal again); group IV (*n* = 5) - 4 weeks 0.5% CED (re-supplementation) + atorvastatin (2.5 mg/kg body weight/day), afterwards 6 weeks SC (~withdrawal again) + atorvastatin (Fig. 1). Animals were sacrificed from 07:00 AM to 10:00 AM, by cervical dislocation as described previously [24] according the recommendation of the working community of the Animal Welfare Officers, Germany.

This investigation conforms to the Guide for the Care and Use of Laboratory Animals (8th edition), 2011 [25]. All animal studies were performed in compliance with the German laws relating to the conduct of animal experimentation. The study (No. Da 5/41) was approved by the regional council Darmstadt, Dept. for veterinary medicine and consumer protection. Our manuscript adheres to the ARRIVE guidelines (http://www.nc3rs.org.uk/page.asp?id=1357) for the reporting of animal experiments.

### Determination of blood lipid levels

Blood was periodically drawn at 1, 6, 14, 21, 29, 40, 74, 76, 78, 80, 82 and 84 weeks as well as at the end of the experiment (sacrifice), from the ear vein using ethylenediaminetetraacetic acid (EDTA) tubes to determine total cholesterol, triglyceride and phospholipids levels using commercially available test kits (Merck, Darmstadt; Biomed, Munich; Wako, Neuss, Germany).

### Aortic tissue of NZW rabbits

The rabbits were sacrificed at the end of the experiment according to the schedule (Fig. 1). For a more precise quantification and documentation of the distribution of atherosclerotic lesions along the complete thoracic aorta, the thoracic aorta was proximo-distally divided into 10 equally sized segments (1–10). Moreover, each of these segments was divided into three sub-segments: the proximal one was embedded in methacrylate, the middle one was shock-frozen in liquid nitrogen-cooled isopentane and stored at − 70 °C until used (for immunohistology and morphometry), and the distal one was snap-frozen in liquid nitrogen and stored at − 20 °C (for biochemical analyses).

### Determination of cholesterol content in segments of the thoracic aorta

The distal part of the third aortic sub-segments was minced, extracted by the Folch procedure [26] and total cholesterol was determined using available test kits (Roche, Mannheim, Germany).

### Immunohistology

Immunohistology was performed as previously described [24, 27]. Immunoreactions were achieved with monoclonal antibodies (mAbs) directed against α-actin (DAKO, Glostrup, Denmark), superoxide dismutase 2 (SOD2), or integrin subunit alpha M (Mac-1, Roche, Mannheim, Germany). Mouse anti-human major histocompatibility complex, class II, DR Alpha (HLA-DRA, LN3 clone, Roche Mannheim, Germany) and mouse anti- tumor protein P53 (Tp53, DAKO), overnight at RT. After washing in phosphate-buffered saline (PBS), the sections were incubated with biotinylated anti-mouse IgG streptavidin-peroxidase (HRP) conjugated (Amersham, Braunschweig, Germany); afterward staining reaction was performed by adding a diaminobenzidine (DAB) solution (Pierce, Rockford, IL, USA), and stained with Mayer’s hematoxylin. From the histological and immunohistological sections, digitalized images were obtained using an Axioplan2 imaging microscope (Carl Zeiss GmbH, Jena, Germany) and the digital high-resolution imaging system AxioCam/AxioVision (Carl Zeiss GmbH).

### Detection of DNA fragmentation (TUNEL technique)

DNA fragmentation was studied on 4% paraformaldehyde-fixed cryo cross-sections by the TUNEL (TdT-mediated dUTP nick end labeling) technique, using the ApopTag kit (Oncor, Heidelberg, Germany) as previously described [24, 27].

### Morphometry

Microscopic images were used to quantify the plaque area and the number of cells (hematoxylin positive nuclei) in cross-sections of the proximal sub-segments of the thoracic aorta. The plaque areas were measured by tracing the internal elastic lamina and the luminal circumference [28]. Total density or the density (n/mm^2^) of immunoreactive (ir) cells in the intima of single-stained sections were determined. Cells were counted when an immunoreactive cell was associated with a nucleus.

### Statistical analyses

Statistical analyses were performed using SigmaPlot®-12 software (Systat Software GmbH, Erkrath, Germany). After testing for normality (by Shapiro-Wilk) and equal variance, one-way analysis of variance (ANOVA) was used. Statistic post hoc analysis was performed using all pairwise multiple comparison procedures according Holm-Sidak. In the case of the normality test failed (*p* < 0.050) ANOVA on ranks begun Kruskal-Wallis test and afterward as post hoc analysis, Dunn’s test was performed. Data are shown as mean + SEM. Differences were considered to be statistically significant when P was less than 0.05. Data correlation was performed using the Pearson’s coefficient analysis.

## Results

### Plasma lipid levels

**Fig. 2:**
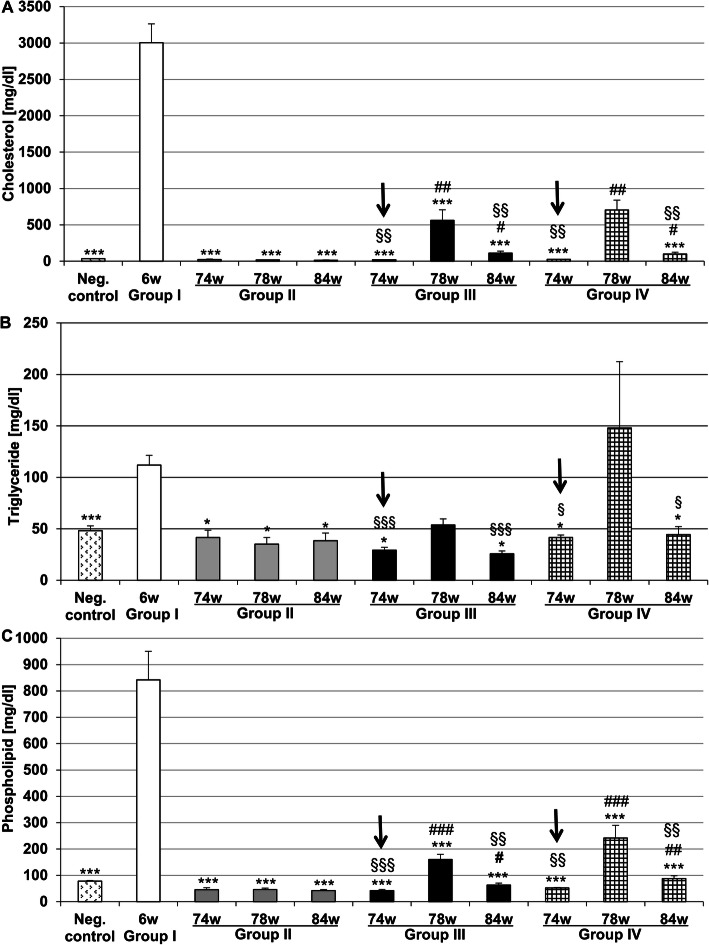
Plasma cholesterol (**a**), triglyceride (**b**) and (**c**) phospholipid levels (in mg/dl) of rabbits of negative control group (neg. control), as well as groups I, II, III, and IV. Black arrows indicate the CEDrs point. Data are expressed as means + SEM; *p < 0.05, **p ≤ 0.01, ***p ≤ 0.001 vs. group I (6 weeks CED, [6w]); ^#^*p* ≤ 0.05, ^##^*p* ≤ 0.01, ^###^*p* ≤ 0.001 vs group II at the corresponding time point; ^§^*p* ≤ 0.05, ^§§^*p* ≤ 0.01, ^§§§^*p* ≤ 0.001 vs 78 weeks (78w); *n* = 5–8

Control (~6 weeks with SC) animals displayed plasma cholesterol levels of 34.6 mg/dl, whereas, after application of a 2% CED for 6 weeks, these levels were significantly (*p* ≤ 0.001) 86.7-fold increased in animals of group I (Fig. 2a); however, after 68 weeks withdrawal (group II; 74 weeks cumulative experimental time) of the CED, groups II-IV showed similar plasma cholesterol levels as the control group (Fig. 2a). At week 84 (i.e. after CED cessation for 78 weeks), group II revealed plasma cholesterol levels similar to control animals (Fig. 2a). 0.5% CEDrs for 4 weeks (at week 78 cumulative time) resulted in a 27.6-fold (*p* ≤ 0.01) and 28.2-fold increase in plasma cholesterol concentration in groups III and IV, in comparison with the preceding time point (74 weeks), i.e. before starting the re-supplementation (Fig. 2a); however, after CED cessation for 6 weeks, the plasma cholesterol levels of groups III and IV (+ atorvastatin) were 5.0- and 7.1-fold reduced, respectively, in comparison with the preceding time point (~ 78 weeks cumulative experimental time, Fig. 2a).

After 6 weeks application of a 2% CED (group I), plasma triglyceride levels significantly (*p* ≤ 0.001) 2.3-fold increased, compared with the control (Fig. 2b). In group II, plasma triglyceride levels significantly (*p* ≤ 0.05) decreased to control values after 74, 78 and 84 weeks (Fig. 2b). In group III after 74 weeks a significant (*p* ≤ 0.01) 1.8-fold increase and 2.1-fold decrease after 84 weeks compared with 78 weeks of plasma triglyceride levels was observed (Fig. 2b); treatment with atorvastatin for 4 weeks together with CEDre (group IV, 78 weeks) resulted in a significant (*p* ≤ 0.05) 3.3-fold increase compared with group IV at 74 weeks and after 6 weeks cessation with atorvastatin treatment yielding the level of the control (Fig. 2b).

In group I, plasma phospholipid levels were significantly (*p* ≤ 0.001) 10.8-fold increased compared with the control group, but significantly (*p* ≤ 0.001) decreased after cessation of the CED (groups II, III and IV, 74 weeks; Figs. 1 and 2c) and remained in group II at “control” level until the end of the experiment after 84 weeks (Fig. 2c). After 4 weeks CEDrs (78 weeks cumulative time) group III showed a significant (*p* ≤ 0.001) 3.8-fold increase in plasma phospholipid level compared with the level at 74 weeks (Fig. 2c); after 6 weeks withdrawal (84 weeks) a significant (*p* ≤ 0.001) 2.5-fold decrease of plasma phospholipid level was found when compared with the level at 78 weeks (Fig. 2c). Group IV (at 74 weeks) showed no difference in plasma phospholipid level compared with group II at 74 weeks, but a significant (*p* ≤ 0.001) 4.6-fold increase after 4 weeks CEDrs (group IV-78 weeks, Fig. 2c); however, after 6 weeks withdrawal of CED (84 weeks cumulative time) the phospholipid level significantly (*p* ≤ 0.001) decreased 2.8-fold compared with group IV (at 78 weeks, Fig. 2c).

### Cholesterol content of the thoracic aorta, plaque size and cell density of plaques

**Fig. 3:**
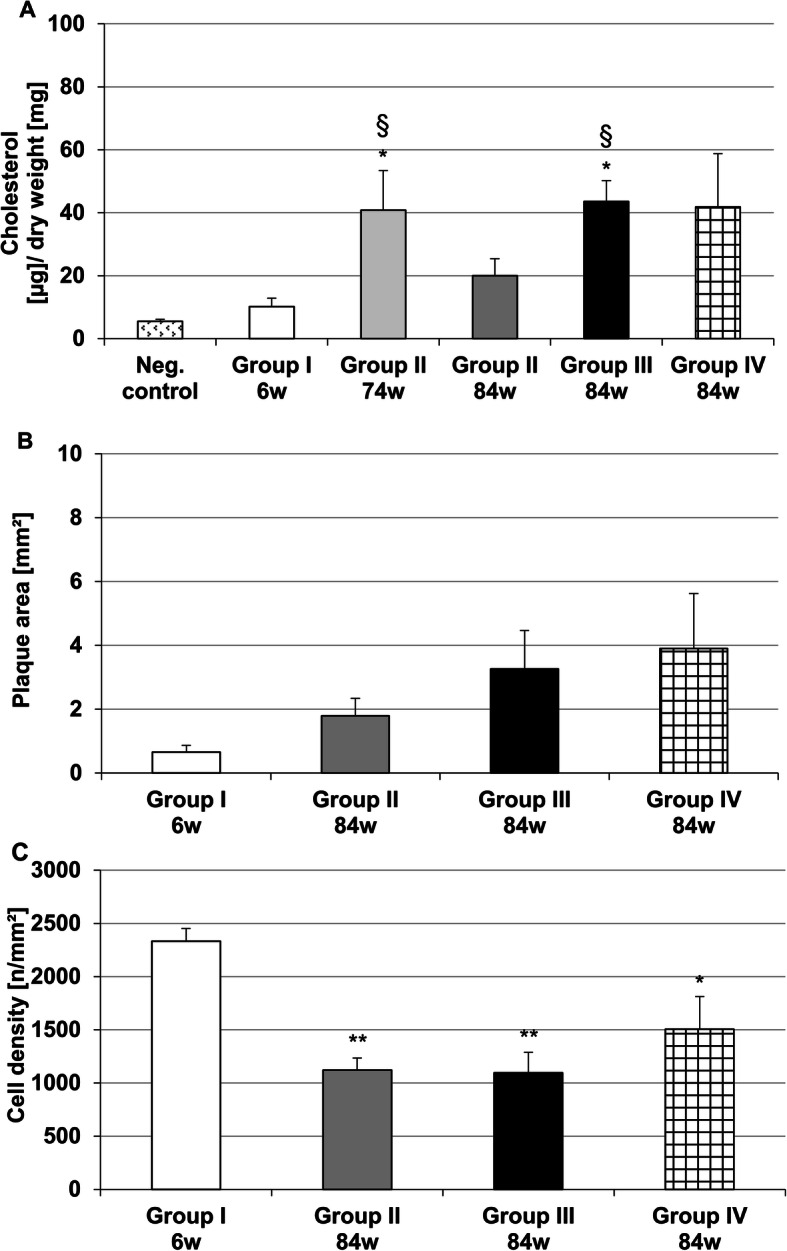
Cholesterol content in proximal segment of the thoracic aorta (**a**) of groups under test. Plaque area [mm^2^] in the thoracic aorta (**b**) and cell density [n/mm^2^] in atherosclerotic plaques (**c**) of the experimental groups. Data are expressed as means + SEM; *p < 0.05, **p ≤ 0.01 vs. group I (6 weeks CED, [6w]); ^§^*p* ≤ 0.05 vs control; *n* = 5–8

Control group revealed cholesterol contents of the thoracic aorta of 5.5 μg/mg dry weight, whereas in group I, after 6 weeks CED, the cholesterol content of the thoracic aorta was almost 1.9-fold higher (Fig. 3a). However, in group II at 74 weeks (68 weeks after cessation of CED) the cholesterol content was significantly (*p* ≤ 0.05) 7.5-fold higher than in the control group and significantly (*p* ≤ 0.05) 4.0-fold higher than in group I (Fig. 3a). Thereafter, in group II at 84 weeks (78 weeks after cessation of CED) cholesterol content of the thoracic aorta was 50% lower than in the same group at 74 weeks. However, in groups III (without atorvastatin) and IV (with atorvastatin) at 84 weeks cholesterol content of thoracic aorta remained 8.0-fold (*p* ≤ 0.05) increased in comparison with the control (Fig. 3a). The cholesterol content of the thoracic aorta of group III (at 84 weeks) was 2.0-fold higher than group II (at 84 weeks, Fig. 3a).

In all segments of the thoracic aorta of the control group, no atherosclerotic lesions were found (not shown). However, after 6 weeks of CED (group I) only small plaques were seen (Fig. 3b). After CEDrs (at 84 weeks), groups III and IV revealed larger plaque areas than groups I and II, however, the differences were not significant (Fig. 3b). Cell densities in atherosclerotic lesions of the groups II, III and IV were significantly (*p* ≤ 0.01; *p* ≤ 0.01 and *p* ≤ 0.05) about 52.0, 53.0% or 35.5% lower when compared with group I, but in groups II and III cell densities (1121 cells/mm^2^ or 1097 cells/mm^2^) were not significantly different from those in group IV (Fig. 3c).

### Cellular composition/morphology of atherosclerotic plaques of the thoracic aorta

**Fig. 4:**
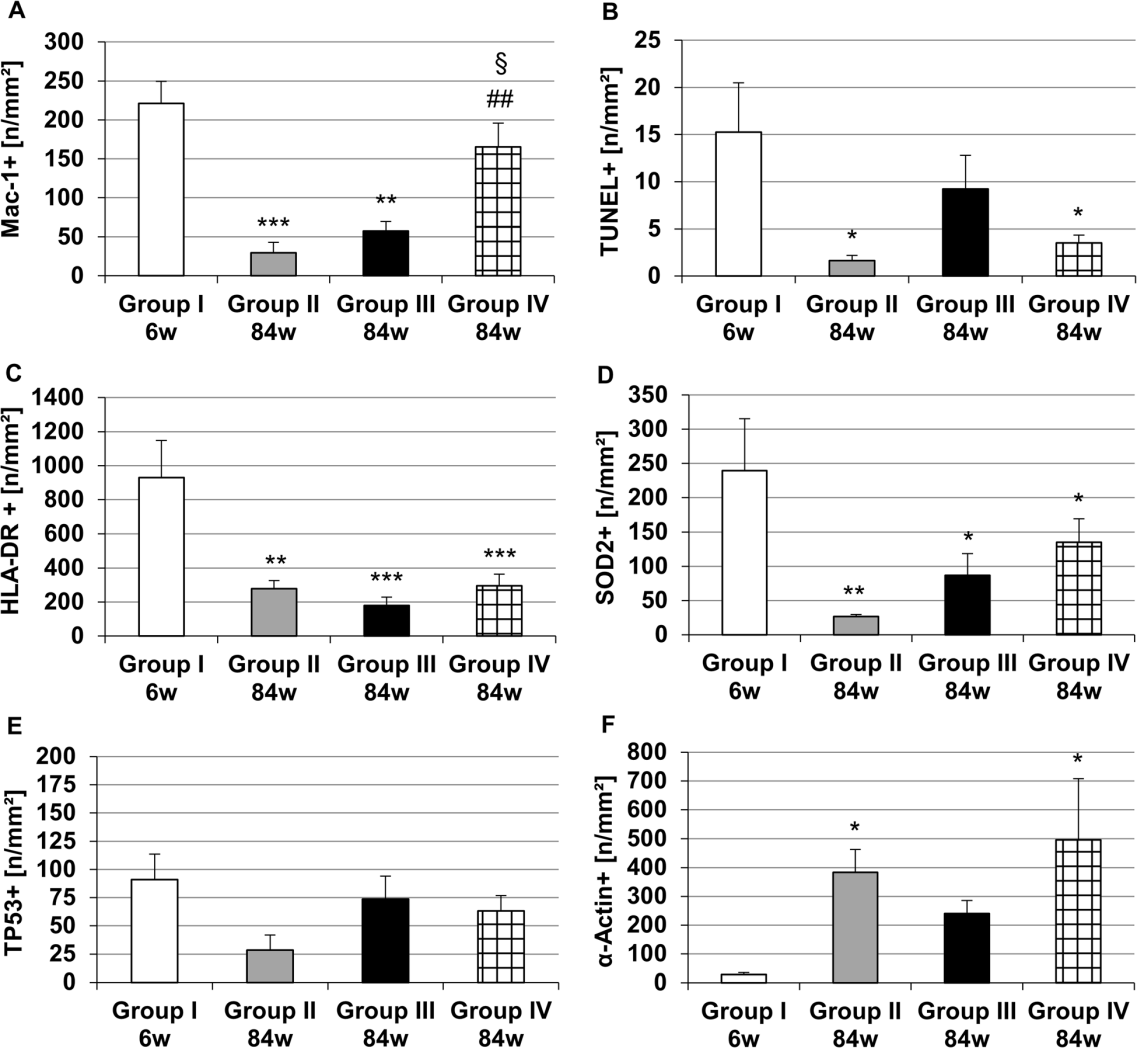
Effect of the CEDrs (at 84 weeks of the experiment) on density of (**a**) MΦ (Mac-1^+^), (**b**) apoptotic cells (TUNEL^+^), (**c**) antigen presenting cells (HLA-DR^+^), (**d**) oxidatively stressed cells (SOD2^+^), (**e**) apoptotic cells (TP53^+^) and (**f**) smooth muscle cells (α-actin^+^) in atherosclerotic plaques of thoracic aortas compared with group I (after 6 weeks induction with CED). Data are expressed as Mean + SEM. *P < 0.05, **P < 0.01, ***P < 0.001 vs. group I; ^##^*P* < 0.01 vs group II; ^§^*P* < 0.05 vs group III. *n* = 5–8

In early lesions, as observed after 6 weeks CED in group I, high numbers of Mac-1^+^ MΦ were found (Fig. 4a). After 84 weeks (78 weeks CED withdrawal) in atherosclerotic lesions of group II, the density of Mac-1^+^ MΦ was significantly (*p* ≤ 0.001) 7.4-fold lower than in group I (Fig. 4a). In atherosclerotic plaques of group III at 84 weeks (after 4 weeks CEDrs and afterward 6 weeks cessation) we found an insignificant 1.8-fold increase of Mac-1^+^ MΦ in comparison with group II, which however was still significantly (*p* ≤ 0.01) 4.0-fold lower than in group I (Fig. 4a). Moreover, CEDrs plus atorvastatin application (group IV) resulted in a further, significant (*p* ≤ 0.01) 5.3-fold increase in density of Mac-1^+^ MΦ in comparison with group II, and a significant (*p* ≤ 0.05) 2.9-fold increase in density of Mac-1^+^ MΦ in comparison with group III (CEDrs without atorvastatin, Fig. 4a).

In lesions of group I, the density of apoptotic (TUNEL^+^) cells was significantly (*p* ≤ 0.05) 9.4-fold or 4.3-fold higher than in group II or IV (Fig. 4b). CEDrs plus atorvastatin application (group IV, 84 weeks) lead to insignificant differences when compared with the groups II and III (without atorvastatin, Fig. 4b).

In atherosclerotic lesions of group I the density of HLA-DR^+^ cells was significantly 3.3- (*p* ≤ 0.01), 5.2- (*p* ≤ 0.001) or 3.1-fold (*p* ≤ 0.001) higher than in the groups II, III, and IV (Fig. 4c), but insignificant differences were observed among group IV (plus atorvastatin), and groups II or III (without atorvastatin, Fig. 4c).

According to SOD2, as an indicator for oxidative stress, we found that in group I density of SOD2^+^ cells was significantly 9.0-fold (*p* ≤ 0.01), 2.8-fold (*p* ≤ 0.05) or 1.8-fold (*p* ≤ 0.05) higher than in the groups II, III, and IV (Fig. 4d), but no significant differences were observed between group IV (plus atorvastatin) and group III (without atorvastatin, Fig. 4d).

The four experimental groups revealed no significant differences in the density of TP53^+^ cells; additionally, no atorvastatin effect was observed concerning the density of TP53^+^ cells (Fig. 4e).

In early atherosclerotic lesions of group I only a very small number of α-actin^+^ SMC could be detected, but after cessation of CED, in groups II and IV (84 weeks) the density of α-actin^+^ SMC was significantly (*p* ≤ 0.05) 13.6-fold and 17.6-fold (*p* ≤ 0.05) higher than in group I, respectively (Fig. 4f). Atorvastatin treatment had no significant effect on the density of α-actin^+^ SMC (Fig. 4f).

### Correlation analyses of cellular markers responsible for cellular composition/morphology of atherosclerotic plaques of the thoracic aorta of groups II, III and IV

Using a Pearson’s correlation coefficient to characterize the cellular plaque composition/morphology in more detail and to predict a possible vulnerability pattern inside the groups, we found definite differences between the groups II and III compared with group IV (Table 1). First of all, cell density did only correlate with the density of Mac-1^+^ MΦ of group I (Table 1).

**Table 1:** Correlation analyses

		Mac-1^**+**^			
		(Group I)	(Group II)	(Group III)	(Group IV)
**Cell Density**	r	**0.95**	0.32	0.78	0.58
	p≤	**0.0486**	0.6800	0.1190	0.2320
	n	**5**	5	5	6
**TUNEL**^**+**^	r	−0.88	0.69	0.59	**0.82**
	p≤	0.1210	0.3140	0.2960	**0.0479**
	n	5	5	5	**6**
**HLA-DR**^**+**^	r	0.87	0.48	**0.92**	**0.84**
	p≤	0.1270	0.5220	**0.0283**	**0.0346**
	n	5	5	**5**	**6**
**SOD2**^**+**^	r	0.56	0.18	0.86	**0.92**
	p≤	0.4440	0.8230	0.0605	**0.0096**
	n	5	5	5	**6**
**TP53**^**+**^	r	0.82	0.91	0.80	**0.84**
	p≤	0.1850	0.0919	0.1010	**0.0349**
	n	5	5	5	**6**

**Fig. 5:**
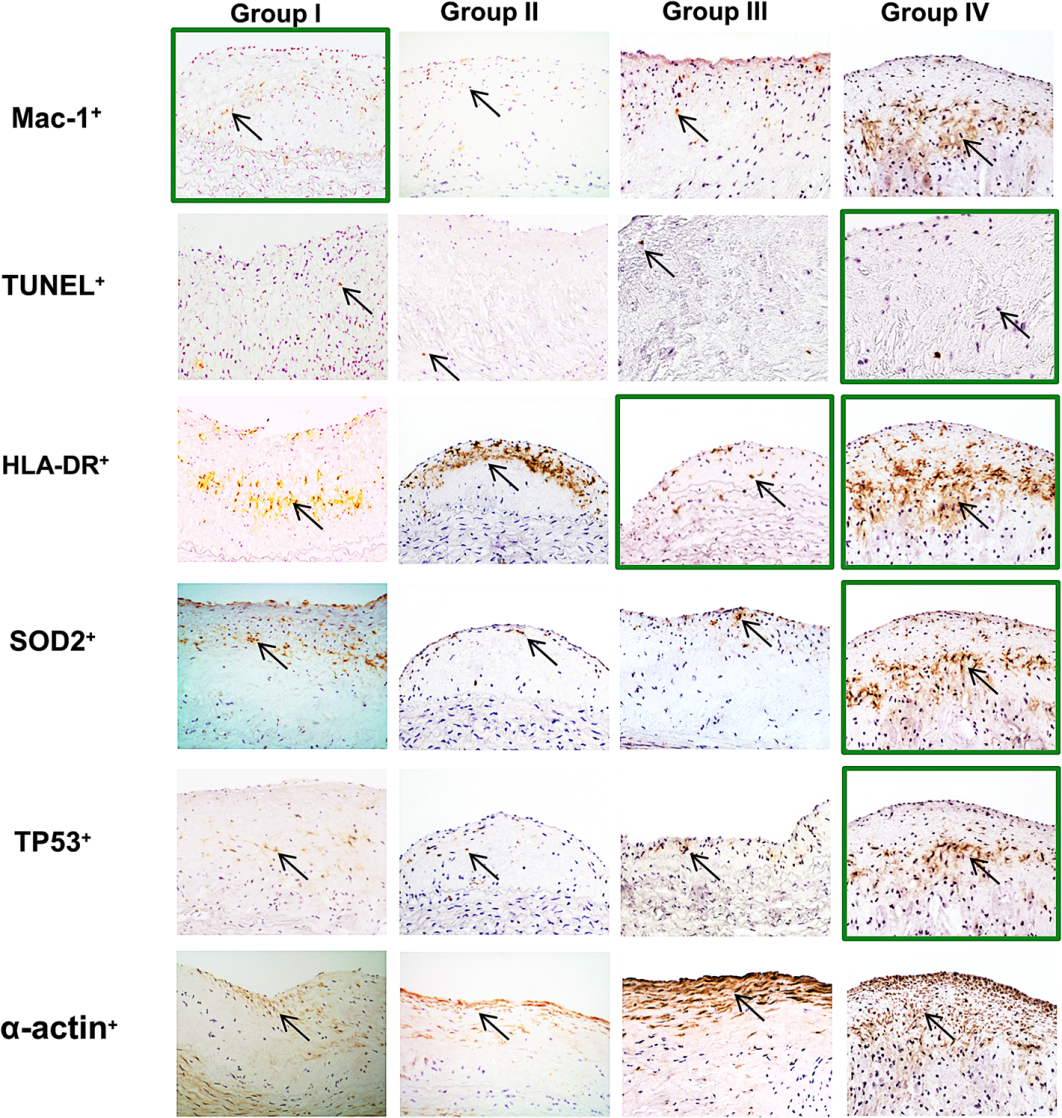
Representative microphotographs of atheromatous lesions of aortic wall sections of groups under test. Black arrows indicate Mac-1 MΦ, TUNEL+, HLA-DR+, SOD2+ or TP53+ immunoreactivity and TUNEL-positive cells. Magnification: × 200

Pearson’s correlation coefficient between the density of Mac-1^+^ MΦ vs densities of TUNEL^+^, HLA-DR^+^, SOD2^+^ or TP53^+^ cells. Data used for the correlation analyses were the mean of the (immuno) histomorphometrical quantifications. The significant correlations were highlighted in bold and correspond to the images framed with green in Fig. 5. Abbreviations: *r*, Correlation coefficient; *p, P*-value; *n*, Number of animals

Additionally, regression analyses of the density of cellular markers responsible for cellular composition/morphology of atherosclerotic plaques revealed that most of the correlations among the cellular markers of lesions in groups II or III (CEDrs) were not significant, with the exception of one significant (*p* ≤ 0.028) positive correlation (*r* = 0.92) between densities of HLA-DR^+^ cells and Mac-1^+^ MΦ in group III (Table 1). However, in group IV (with atorvastatin treatment), we found significant positive correlations between density of Mac-1^+^ MΦ and apoptotic (TUNEL^+^) cells (*r* = 0.82, *p* ≤ 0.048), Mac-1^+^ MΦ and antigen-presenting (HLA-DR^+^) cells (*r* = 0.84, *p* ≤ 0.035), Mac-1^+^ MΦ and oxidatively stressed (SOD2^+^) cells (*r* = 0.92; *p* ≤ 0.0096) or Mac-1^+^ MΦ and TP53^+^ (*r* = 0.84, *p* ≤ 0.035) cells (Table 1). Correlation analyses correspond to the representative images enclosed in Fig. 5, where the localization of Mac-1^+^ MΦ in lesions of group IV co-localizes with TUNEL^+^, HLA-DR^+^, SOD2^+^ or TP53^+^ immunoreactivity (Fig. 5).

## Discussion

The prevention and treatment of CVD remain an incontrovertible fact by reducing the CV risk factors such as increased lipid or systolic blood pressure promoting health by a change of an unhealthy lifestyle, e.g. lack of physical activity, poor-quality diet, overweight, smoking, as well as elevated blood cholesterol levels. Because a high blood cholesterol level is an independent cardiovascular risk factor, its lowering is a major goal of an anti-atherosclerotic therapy [3, 29, 30]. Patients with an established CVD or with an increased risk of developing CVD, necessarily need to reduce their blood cholesterol levels by using medication, e.g. statins. However, nobody knows the consequences of varying cholesterol levels or statin treatment on plaque size and influence on the cellular composition of already existing plaques. Thus, and because rabbits are widely accepted for the study of human atherosclerosis [31] since they are sensitive to a cholesterol diet and have similar features of lipoprotein metabolism, like humans but unlike rodents [31], the purpose of our study, by using rabbits with pre-lesioned thoracic aortas, was to investigate the effects of a CEDrs with or without statin treatment on plaque size and/or cellular composition. We found that re-supplementation for 4 weeks resulted in a significant increase in plasma cholesterol levels, whereas afterward withdrawal of CED for 6 weeks reduced plasma cholesterol levels to almost the levels before starting the re-supplementation. In this context, it has most recently been shown, that in rabbits – after application of a 0.5% CED – a treatment with atorvastatin (2 mg/kg/day) for 10 weeks, did also not significantly reduce the absolute LDL-C and triglyceride levels in comparison with the control at distinct time points [32]. However, the same study revealed that treatment of atorvastatin significantly reduced the lesion size by 68% [32], whereas our data reveal that after CEDrs and withdrawal of CED (group III), plaque area is not significantly different from the group without re-supplementation (group II), despite an increased cholesterol content of the vessel wall; however, in our re-supplemental study (group IV), atorvastatin treatment did neither affect plaque size nor cholesterol content in comparison with the group without atorvastatin (group III) treatment. Thus, atorvastatin has different effects on inhibition of plaque development/progression depending on, whether the lesions already exist or just develop. Moreover, CEDrs (group III) did not significantly influence cell density, and atorvastatin treatment (group IV) did not as well. While the regression of advanced atherosclerotic plaques after normalization of pro-atherogenic factors in rodents and non-human mammals, as well as in patients, is differently discussed [24, 33–35], the cellular reorganization of the plaque is suggested to lead to varying plaque stabilities [16]. In our experiments, we have used the lowest dose as suggested by others [36]. We assume that the atorvastatin dose was too low and / or the four-week application time was too short to see a more obvious effect on vessel morphology. In this context, the density of MΦ may also be an important factor for the regression of atherosclerosis, as demonstrated in rabbits [11, 18, 37]. In respect thereof, we found characteristic signs of plaque remodelling as an increase of the presence of MΦ after CED induction (group I) or atorvastatin treatment after CEDrs (group IV), but not in group II (long-term withdrawal) or group III (short-term withdrawal after 4 weeks CEDrs). Additionally, we found a reduced number of apoptotic cells in atherosclerotic lesions of groups II and IV, which may evidence that MΦ are still necessary for the plaque remodelling after atorvastatin treatment [11]. This is necessary to mention because in human atherosclerotic lesions apoptosis was abundantly found and contributed to the accumulation of gruel and plaque instability [38] and macrophage death may be a promising pharmacological target in atherosclerosis [39]. Moreover, we found similar densities of HLA-DR^+^ cells in groups II, III and IV with a significant positive correlation between the density of Mac-1^+^ MΦ and HLA-DR^+^ cells, indicating that density of antigen-presenting cells (APC) is affected neither by CEDrs nor by atorvastatin treatment. However, APC localized in atherosclerotic plaques of the thoracic aortas of rabbits under test are suggested to be MΦ. As we also found the highest density of SOD2^+^ cells in the plaques of group I (induction), this may indicate that oxidative stress in MΦ, possibly induced by oxidized low-density lipoprotein (oxLDL), may cause apoptosis and in terms of antigen presentation, attract more phagocytes from the blood stream [40]. Since administration of atorvastatin induces plaque stabilization [32] and an absence of TP53 accelerates atherosclerosis [41], it is of interest, that we found a significant positive correlation between the density of Mac-1^+^ MΦ and density of TP53^+^ cells, which may be an indicator of improved plaque stabilization as found for lesions exclusively in group IV (with atorvastatin treatment). Accordingly, simvastatin was shown to stabilize or even regress established atheromatous lesions in rabbits at a dose rate of 7 mg/kg/day [42], which was about 3-fold higher than the atorvastatin dose rate in our experiment. In this respect, 4 weeks of CEDrs and additional withdrawal for 6 weeks (Group III), resulted in a decreased density of MΦ and an increased density of α-actin^+^ SMC compared with group I. Excess stimulation of SMCs by mitogens and secretion of the vessel wall components is assumed to contribute to plaque growth and might explain the lack of regression in some species under certain pathophysiological conditions [43]. In agreement with our previous observations, after the withdrawal of CED for up to 1.5 years, neither regression nor progression of the lesion size was observed, whereas the atherosclerotic plaques respond by a decrease in the number of MΦ and an increase of SMCs [24]. According to other publications, coronary plaque stabilization was found in parallel with a decrease of lipid and MΦ content, as well as impaired migration of SMCs in hypercholesterolemic miniature pigs [11, 44]. Our data show, that the composition, but not the size of atherosclerotic plaques, respond to lowering of cholesterol levels, suggesting their reorganization to more stable plaques. It may be reasonable to assume that a complete cessation of fat consumption and statin treatment would not heal, reduce and/or stabilize the atherosclerotic lesion or arrest the further progression of the disease process, as we have shown after short-term statin treatment and CED interruption. In humans, this might be the case, e.g. if the patients abandon their treatment and discontinue a healthy lifestyle prior to reaching their therapeutic effect. However, this negative attitude can be more or less risky which depends on the length of time between the interruption phase and the re-establishment of the treatment as well as a healthy lifestyle. Nevertheless, it is unclear if these effects on the plaque morphology and quality occur in animals as well as in humans [45]. Although, an increase of cholesterol concentration in the vessel wall (Gruppe IV) is suggested to be responsible for the activation of plaque MΦ leading to e.g. oxidative stress and apoptosis, as demonstrated in the present study. Atorvastatin treatment seems to activate conditions for plaque remodeling and to accelerate the stabilization compared with the group without atorvastatin treatment. In this context, in vulnerable plaques of humans, which present a thin fibroatheroma cap and a lipid core with macrophage infiltration, statins increased the fibrous cap thickness and reduced the lipid core, which may be interpreted as a process of plaque stabilization [46].

We suggest, that either the dose of atorvastatin was too low and/or the application time of 4 weeks was too short. Moreover, high-intensity atorvastatin therapy, 80 mg daily for 24 months, has been demonstrated to promote coronary atheroma stabilization and regression [47, 48]. Statin treatment was in direct association with changes in atheroma composition, reducing fibro-fatty components [47]. However, atherosclerosis continues to progress [49] or not retreats as happens in our study with rabbits. In this context, new therapeutic strategies must use a combination of treatments, e.g. dietary reduction of lipids and carbohydrate intake, together with statins and agents to inhibit cholesterol absorption (*e. g.* ezetimibe) or proprotein convertase subtilisin/kexin type 9 (PCSK9) activity (*e. g.* evolocumab) [50]. Thus, it is noteworthy to mention, that recent investigations have shown a relationship between serum PCSK9 levels and cardiovascular events as e.g. myocardial infarction, revascularization, thrombotic stroke [51]. PCSK9 released by endothelial and smooth muscle cells may have paracrine influence, on the expression of LDLR on macrophages and induce its differentiation in foam cells within atherosclerotic plaques [52]. In patients with cardiovascular disease, PCSK9 blood concentrations were related to the frequent presence of necrotic cores in coronary plaques regardless of statin use [53]. Unfortunately, the PCSK9 inhibition on plaque regression is unknown [54]. In this regard, by using the experimental animal model of our study, it would be an interesting perspective to investigate the influence of PCSK9 on cholesterol levels and plaque morphology, as a potential promising target for new therapies, together with the reduction of dietary intake of lipids.

According to our results, long-term plaque reorganization suggests a predisposition to plaque stabilization due to normalized pro-atherogenic risk factors, like cholesterol levels. In contrast, cholesterol re-supplementation seems to induce a development of a more vulnerable plaque, but the re-withdrawal modifies the plaque to a more stable state. Consequently, the regression of atherosclerosis unfortunately depends on the unpredictable development of atherosclerotic lesion, which depends at the same time on the individual variability, as well as the history of the disease of each patient.

Therefore, further CEDrs studies – using rabbits with pre-lesioned thoracic aortas - with withdrawal period longer than 6 weeks are needed to investigate, whether the withdrawal time and/or the dose and/or time of atorvastatin treatment improve the plaque stability and/or definitely induce its regression partially or even completely. In order to define processes, which promote or reduce the development either of destabilizing or stabilizing plaque structures, further studies are needed, especially with regard to the development of atherosclerotic lesions in rabbits/patients with long-term dietary exogenous dyslipidemia, including prolongated withdrawal time and lipid-lowering drugs as well. According to our results, we suggest, that the period of time/concentration of statin treatment and/or regression of blood cholesterol levels may influence the curability of patients by modulating the grade of stability of atherosclerotic plaques; otherwise non-curable patients will land up at a point of no return.

## Conclusions

The regression of the atherosclerosis disease depends on the unpredictable development of the atherosclerosis lesion, which depends at the same time on the individual variability, as well as history of the disease of each patient. According to our results we suggest, that the period of time/concentration of statin treatment and/or blood cholesterolemia regression may influence the curability of patients by modulating the grade of stability of atherosclerotic plaques; otherwise non-curable patients land up at a point of no return. Therefore, further CEDrs studies – using rabbits with pre-lesioned thoracic aortas - with withdrawal period for more than 6 weeks are needed to investigate, whether the withdrawal time and/or the atorvastatin treatment improve the plaque stability and/or definitely induce its regression in part.

## Data Availability

The datasets that support the findings of this study are available from the corresponding author on reasonable request.

## Abbreviations

ANOVA: Analysis of variance
APC: Antigen-presenting cells;
CED: Cholesterol-enriched diet
CVD: Cardiovascular diseases
DAB: Diaminobenzidine
EDTA: Ethylenediaminetetraacetic acid
HLA-DRA: Major histocompatibility complex, class II, DR alpha
HRP: Horseradish peroxidase
Ir: Immunoreactive
LDL: Low-density lipoprotein
Mac-1: Integrin subunit alpha M
MΦ: Macrophage(s)
mAbs: Monoclonal antibodies
NZW rabbit: New Zealand white rabbit
oxLDL: Oxidized low-density lipoprotein
PBS: Phosphate-buffered saline
PCSK9: Proprotein convertase subtilisin/kexin type 9
RT: Room temperature
SC: Standard chow
SMC: Smooth muscle cells
SOD2: Superoxide dismutase 2
TP53: Anti- tumor protein P53
TUNEL: TdT-mediated dUTP nick end labeling.
